# Generation of Ca^2+^-independent sortase A mutants with enhanced activity for protein and cell surface labeling

**DOI:** 10.1371/journal.pone.0189068

**Published:** 2017-12-04

**Authors:** Hee-Jin Jeong, Gita C. Abhiraman, Craig M. Story, Jessica R. Ingram, Stephanie K. Dougan

**Affiliations:** 1 Department of Cancer Immunology and Virology, Dana-Farber Cancer Institute, Boston, Massachusetts, United States of America; 2 Department of Microbiology and Immunobiology, Harvard Medical School, Boston, Massachusetts, United States of America; 3 Department of Biology, Gordon College, Wenham, Massachusetts, United States of America; University of Texas Medical School at Houston, UNITED STATES

## Abstract

Sortase A, a calcium-dependent transpeptidase derived from *Staphylococcus aureus*, is used in a broad range of applications, such as the conjugation of fluorescent dyes and other moieties to proteins or to the surface of eukaryotic cells. *In vivo* and cell-based applications of sortase have been somewhat limited by the large range of calcium concentrations, as well as by the often transient nature of protein-protein interactions in living systems. In order to use sortase A for cell labeling applications, we generated a new sortase A variant by combining multiple mutations to yield an enzyme that was both calcium-independent and highly active. This variant has enhanced activity for both N- and C-terminal labeling, as well as for cell surface modification under physiological conditions.

## Introduction

Gram-positive bacteria display proteins at their surface for uptake of nutrients and to execute functions associated with pathogenesis. Sortases, a family of membrane-associated transpeptidases, anchor many of these proteins to the cell surface [1]. In *Staphylococcus aureus*, sortase A (SrtA) recognizes the 5-amino acid motif Leu-Pro-Xxx-Thr-Gly (LPXTG, where Xxx is any amino acid, and glycine cannot be a free carboxylate), at or near the C terminus. SrtA cleaves the scissile bond between threonine and glycine to yield an acyl-enzyme intermediate. This intermediate is then resolved by a nucleophilic attack of an oligoglycine-linked substrate to generate a new peptide bond between the LPXT- and oligoglycine-containing molecules [1]. Borrowing the functionality of sortase from its bacterial origin presents a wide range of possible applications. Sortase-mediated transpeptidation, or “sortagging”, refers to a reaction that uses sortase to ligate an LPXTG-tagged molecule to an oligoglycine nucleophile [2]. SrtA can covalently and site-specifically link fluorescent dyes, carbohydrates and other moieties to protein substrates and to the cell surface [3–5]. Sortagging applications have included the cyclization of proteins and peptides [6], modification and labeling of antibodies [7] and the synthesis of protein conjugates with drugs, peptides, peptide nucleic acids and sugars [8].

Wild type SrtA operates with rather slow kinetics, necessitating prolonged incubation times to obtain acceptable product yields. Several groups have contributed to optimizing the SrtA enzyme to generate variants with increased K_m_ and K_cat_ [9,10]. Of these, a pentamutant (“5M”) variant (P94R/D160N/D165A/K190E/K196T) showed a 140-fold increase in activity over wild-type SrtA, with much improved reaction rates even at low temperature [9]. This SrtA variant has been widely adopted for sortagging applications. To achieve a calcium-independent enzyme, mutations at Glu105 and Glu108 were introduced in *S*. *aureus* SrtA to mimic its *S*. *pyogenes* equivalent; however this version suffered from reduced activity relative to its wild type counterpart [11]. To mitigate this, several groups independently generated heptamutant SrtA variants ("7M SrtA") by combining calcium-independent mutations with 5M SrtA to yield Ca^2+^ -independent versions [12–15]. One such variant has been shown to be rate-enhanced by kinetic measurements [14], whereas other groups have established its efficacy in various applications [12,13,15].

Directed evolution efforts derived from wild-type and 5M SrtA yielded further improved variants with increased conjugation efficiency [10]. A D124G mutation showed a 1.6-fold increase in K_cat_ and a 1.8-fold decrease in K_m_, resulting in a 3-fold improvement in catalytic efficiency over 5M SrtA. Mutations at Tyr187 and Glu189, which lie between the β7/β8 loop of SrtA in the same location as the K190 mutation in 5M, also enhanced enzymatic activity. The inclusion of further mutations at Asp124, Tyr187 and Glu189 to the 5M SrtA variant resulted in 1.1 to 4.6 fold improvement in catalytic efficiency over unmodified 5M SrtA [9,10].

The majority of sortase applications concern its use *in vitro*. Labeling proteins in living systems could be a powerful technique to introduce non-genetically encoded linkages between proteins, or between a protein and a non-biologic entity such as a fluorescent dye. Intracellular sortase labeling has been described using a natural occurring calcium-independent sortase A from *S*. *pyogenes* to achieve protein ligation in *S*. *cerevisiae* and mammalian HEK293T cells, both in the cytosol and in the endoplasmic reticulum (ER) [16]. More recently, *in vivo* protein ligation in *C*. *elegans* and in *E*.*coli* was achieved using 7M sortase variants [14,17]. In each of these cases, the sortase enzyme is expressed within cells, along with proteins bearing the LPETG and GGG motifs, enabling inducible formation of unnatural protein-protein interactions.

Another intriguing application is the use of sortase to covalently link a modified peptide that includes the LPETG sequence to naturally expressed surface-exposed N-terminal oligoglycines present at the cell surface [18,19]. A previous report laid the groundwork for this methodology by conjugating LPETG-tagged probes and proteins to the surface of *S*. *cerevisiae*, murine lymphocytes, 293T cells and *Toxoplasma gondii* using wild-type SrtA, thus showing that a sufficient number of naturally exposed glycines on the cell surface are accessible to sortase for labeling [18]. Cell surface labeling could also be extended to cells engineered to express the LPETG motif on their surface: addition of a GGG-bearing nucleophile allowed the transpeptidation reaction to proceed. Such methodology has been used to engineer “sortaggable” red blood cells for the delivery of therapeutic payloads [19,20]. This results in permanent covalent addition of probes to the surface of target cell populations that can be tracked over long periods of time, across the body of an organism, and visualized by microscopy or flow cytometry.

In order to use *in vivo* sortase labeling to capture transient cell-cell interactions, a sortase mutant that is both calcium-independent and retains high activity is required. A version of sortase able to withstand both high and low concentrations of Ca^2+^ is particularly desirable because of the broad range of physiological calcium concentrations present in different cellular environments. The concentration of free Ca^2+^ ranges from approximately 100 nM in the cytoplasm of cells to 1.4 mM in interstitial fluid [21]. Calcium concentrations in the ER range from 1 to 400 μM in response to depletion and replenishment of calcium stores [21]. Thus, *in vivo* applications ideally would require a sortase mutant that is active across a 10,000-fold difference in calcium concentration. Using *ex vivo* cell labeling experiments as a surrogate, we report the development of a Ca^2+^-independent SrtA mutant that fulfills these criteria. We demonstrate its enhanced sortagging activity over a broad range of calcium concentrations for both protein and cell surface labeling.

## Materials and methods

### Animal procedures

This study was carried out in strict accordance with the recommendations in the *Guide for the Care and Use of Laboratory Animals* of the National Institutes of Health, and approved by the DFCI IACUC Committee on Animal Care (Protocol #14–019). Mice were housed at Dana-Farber Cancer Institute. C57BL/6 mice were purchased from Jackson Labs. Mice were euthanized by asphyxiation with carbon dioxide, and all efforts were made to minimize suffering.

### SrtA variant cloning

7+ and 5+ SrtA variants were ordered as synthetic gBlocks from Integrated DNA Technologies, and amplified by PCR with Phusion High Fidelity Polymerase (New England Biolabs) and the following primer pairs: 53_Forward 5’-GGGAAACATATGCAAGCTAAACCT C-3’; 56_Reverse 5’-CTGCTGCTCGAGTTTGACTTCTGTAG-3’. The resulting products were cloned into pET30b+ (Novagen) with NdeI and XhoI sites. Other SrtA variants were generated by overlapping PCR and Gibson fragment assembly, and cloned into pET30b+. The following primer sets were used: pET30b_GibF 5’-GTTTAACTTTAAGAAGGAGATATAC-3’; pET30b_GibR 5’-TCAGTGGTGGTGGTGGTGGTG-3’; SrtADG_F 5’-CACTTTCATTGGCCGTCCGAAC-3’; SrtADG_R2 5’-GTTCGGACGGCCAATGAAAGTG-3’; SrtAYLER_F 5’-CTTGTGATGATCTGAATCGCGAGACAGGCG-3’; and SrtAYLER_R 5’-CGCCTGTCACGCGATTCAGATCATCACAAG-3’.

### Expression and purification of SrtA mutants

*E*.*coli* BL21(DE3) were transformed with pET30b^+^ containing 5M, 5D, 5Y, 5+, 7M, 7D, 7Y or 7+ SrtA constructs and cultured at 37°C overnight in 5 mL of Luria Broth (LB) media supplemented with 34 μg/ml kanamycin. This was used to inoculate 200 mL of Terrific Broth (TB) media supplemented with 34 μg/ml kanamycin and cultured at 37°C until an OD_600_ ~0.6, at which point 1 mM isopropylthio-**β**-galactopyranoside (IPTG) was added and cultures induced overnight at 30°C. For 5+ SrtA, 0.4 mM IPTG was used, and induction was at 16°C overnight. Cells were harvested by centrifugation (6,000 rpm, 30 min, 4°C) and the resulting pellet was resuspended in 50 mL of Ni^2+^-NTA wash buffer (50 mM Tris, 150 mM NaCl, 10 mM imidazole, pH 7.6) and lysed by sonication. To harvest the soluble fraction, the lysate was again centrifuged (6,000 rpm, 30 min, 4°C) and the resulting supernatant was incubated with 2 mL of Ni^2+^-NTA agarose resin (Qiagen) on a rotating wheel at 4°C overnight. The resin was washed with 30 mL of Ni^2+^-NTA wash buffer and eluted with 5 mL of Ni^2+^NTA elution buffer (50 mM Tris, 150 mM NaCl, 500 mM imidazole, pH 7.6), then buffer exchanged into 50 mM Tris (pH 7.5) in a 3kD molecular weight cut off (MWCO) ultrafiltration device (Millipore). Protein purity was analyzed by SDS-PAGE using a 4–20% polyacrylamide gel (Bio-Rad). The concentration of the protein was calculated using A280 absorbance on a NanoDrop (Thermo). Amino acid sequences, molecular weights and calculated extinction coefficients are listed in S1 Table.

### Protein modeling

Pymol software (The PyMOL Molecular Graphics System, Version 1.8 Schrödinger, LLC) was used to generate the sortase model based on the wild type SrtA (PDB 2kid).

### Expression and purification of substrates

*E*.*coli* WK6 were transformed with vectors encoding camelid single domain antibody fragments (VHHs)—pHEN6-VHH1 or VHH2—and cultured at 37°C overnight in 5 mL of LB supplemented with 100 μg/ml ampicillin). This was used to inoculate 200 mL of TB supplemented with 100 μg/ml ampicillin, and cultured at 37°C until an OD_600_ ~0.6, at which point 1mM IPTG was added and cultures induced overnight at 30°C. Cells were harvested by centrifugation (6,000 rpm, 30 min, 4°C), and the periplasmic fraction was isolated by resuspending pelleted cells in 25 mL of 1X TES buffer (0.2 M Tris, 0.65 mM EDTA, 0.5 M Sucrose) on a rotating wheel at 4°C for 1 hour, and then diluted with an additional 50 mL of 0.25X TES buffer, and incubated overnight at 4°C with agitation. After centrifugation (6,000 x rpm, 30 min, 4°C), the supernatant was incubated with 10 mL of Ni^2+^-NTA agarose resins on a rotating wheel at 4°C for 2 hours. The resins were washed with 100 mL of Ni^2+^-NTA wash buffer and eluted with 15 mL of Ni^2+^-NTA elution buffer (50 mM Tris, 150 mM NaCl, 500 mM imidazole, pH 7.6), then buffer exchanged to 50 mM Tris (pH 7.5) in a 3kD MWCO ultrafiltration device (Millipore). Protein purity was assessed by SDS-PAGE using a 4–20% SuperSep gel, and the concentration of protein was measured by NanoDrop.

### Cleavage assays

Reactions were assembled in a volume of 100 μL containing 5 μM Srt, 50 mM Tris, pH 8, 150 mM NaCl, and either 10 mM CaCl_2_ or 10 mM EGTA with 5, 10, 20, 30, 40 μM Dabcyl-QALPETGEE-Edans (Anaspec). Incubations were carried out at room temperature for 1 h. Samples were analyzed in a fluorometer (SpectraMax M5e, Molecular Devices) using 355 nm for excitation and 538 nm for emission. Reactions were performed in duplicate and averaged, and plotted as arbitrary fluorescent units converted per minute using Prism 7.0.

### Fluorescent labeling of VHHs using SrtA

For C-terminal labeling, 5 μM SrtA and 500 μM GGGK-TAMRA (10 mM stock in DMSO) (Life Technologies) were added to 5 μM VHH1-LPETGG in 50 mM Tris pH 8, 150 mM NaCl and either 10 mM CaCl_2_ or 10 mM EGTA. The resulting mixture was incubated at 4°C for 0–120 min; reactions were stopped by the inclusion of SDS-loading buffer and denaturation at 95°C for 5 min. For N-terminal labeling experiments, 5 μM of GGGGG-VHH2 and 500 μM of Alexa647-LPETGG were used following identical protocols.

### VHH-VHH conjugation using SrtA

5 μM SrtA and 5 μM VHH1-LPETGG were added to the 5 μM GGGGG-VHH2 in 50 mM Tris, pH 8, 150 mM NaCl, and either 10 mM CaCl_2_ or 10 mM EGTA. The resulting mixture was incubated at 4°C for 120 min, then the reaction was stopped by the inclusion of SDS-loading buffer and denaturation at 95°C for 5 min.

### SDS-PAGE analysis

Protein samples were run on SDS-PAGE, then was fixed in 40%(v/v) ethanol and 10%(v/v) acetic acid, prior to staining with Coomassie Brilliant Blue (CBB), then destained in 40%(v/v) ethanol and 10%(v/v) acetic acid. The fluorescence was monitored using a gel scanner gel scanner (ChemiDoc, Bio-Rad). Band intensity of both fluorescent scans and Coomassie staining was quantified using ImageLab software (Bio-Rad). Sortagging efficiency was calculated as the intensity of the dye-labeled VHH as determined by fluorescence scanning of the gel (or the high molecular weight band corresponding to the VHH-VHH fusion) divided by the cumulative intensities of the Coomassie-labeled bands corresponding to the unreacted VHH and the labeled VHH.

### Cell surface labeling

One million spleen cells were isolated from a C57BL/6 mouse [22], and incubated with 1 μM soluble SrtA and 10 μM HA-LPETG in 300 μL RPMI complete media (10% inactivated fetal bovine serum, 100 U/mL penicillin-streptomycin, 1% MEM (Gibco) Non-Essential Amino Acids Solution, 1 mM sodium pyruvate, 1% GlutaMAX, (Gibco) 0.1 mM β-mercaptoethanol) supplemented with 10 mM CaCl_2_ at 37°C for 1 h with agitation. Cells were washed and incubated with APC-conjugated anti-CD8 antibody (BioLegend), BV711-conjugated anti-CD45 antibody (BioLegend), AlexaFluor488-conjugated anti-HA antibody (BioLegend), and Viaprobe (BD Bioscience) at 4°C for 30 min. Cells were then washed, fixed with 0.1% formalin, and visualized by flow cytometry (Sony Spectral Cell Analyzer SP6800). Mean fluorescence intensity was calculated using Sony SP6800 Spectral Analyzer and graphed using Prism. Histograms were produced using FlowJo version 10.0.7.

### Western blot analysis

Spleen cells from a C57BL/6 mouse were incubated at 37°C for 1 h with or without 10 μM HA-LPETG and with or without 1 μM 7+ or 7M SrtA. The reaction mixture was then boiled in SDS loading buffer at 95°C for 10 min, run on a 12% Tris-glycine polyacrylamide gel, and transferred to PVDF. The blot was probed using anti-HA-HRP (Sigma) at a 1:2000 dilution. As a loading control, the blot was stripped and re-incubated with an anti-beta tubulin-HRP antibody (CST) at 1:1000.

## Results

### Generation of sortase A mutants

**Fig 1:**
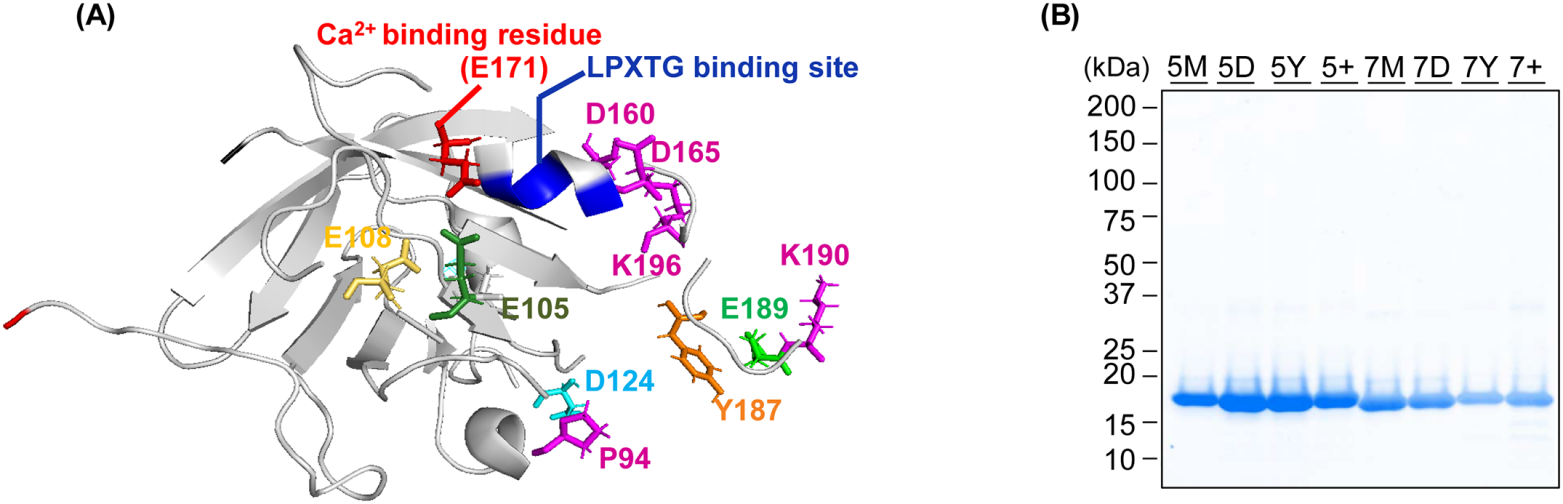
Generation and purification of SrtA variants. (A) Schematic structure of wild type SrtA (PDB 2kid). Calcium-binding residues and LPETG binding site are shown in red and blue, respectively. Mutated amino acids are shown in stick representation. (B) SDS-PAGE gel of bacterially expressed sortase variants following Ni^2+^-NTA purification.

In order to generate a new sortase A mutant useful for cell surface labeling, we sought to combine mutations that conferred calcium independence as well as improved enzyme activity. E105K and E108Q mutations conferring calcium independence were combined with a series of recently described mutations that show enhanced activity in presence of 10mM Ca^2+^ (Table 1, Fig 1A) [10,11]. In all cases, highly purified SrtA mutants were obtained in soluble form at 80–90 mg per 1 L culture (Fig 1B).

**Table 1:** Sortase A variants.

	Mutations	Reference
5M	P94R, D160N, D165A, K190E, K196T	[9]
5D	P94R, D160N, D165A, K190E, K196T, D124G	[10]
5Y	P94R, D160N, D165A, K190E, K196T, Y187L, E189R	[10]
5+	P94R, D160N, D165A, K190E, K196T, D124G, Y187L, E189R	[10]
7M	P94R, D160N, D165A, K190E, K196T, E105K, E108Q	[12,13]
7D	P94R, D160N, D165A, K190E, K196T, E105K, E108Q, D124G	This study
7Y	P94R, D160N, D165A, K190E, K196T, E105K, E108Q, Y187L, E189R	This study
7+	P94R, D160N, D165A, K190E, K196T, E105K, E108Q, D124G, Y187L, E189R	This study

### 7X SrtA mutants are active

**Fig 2:**
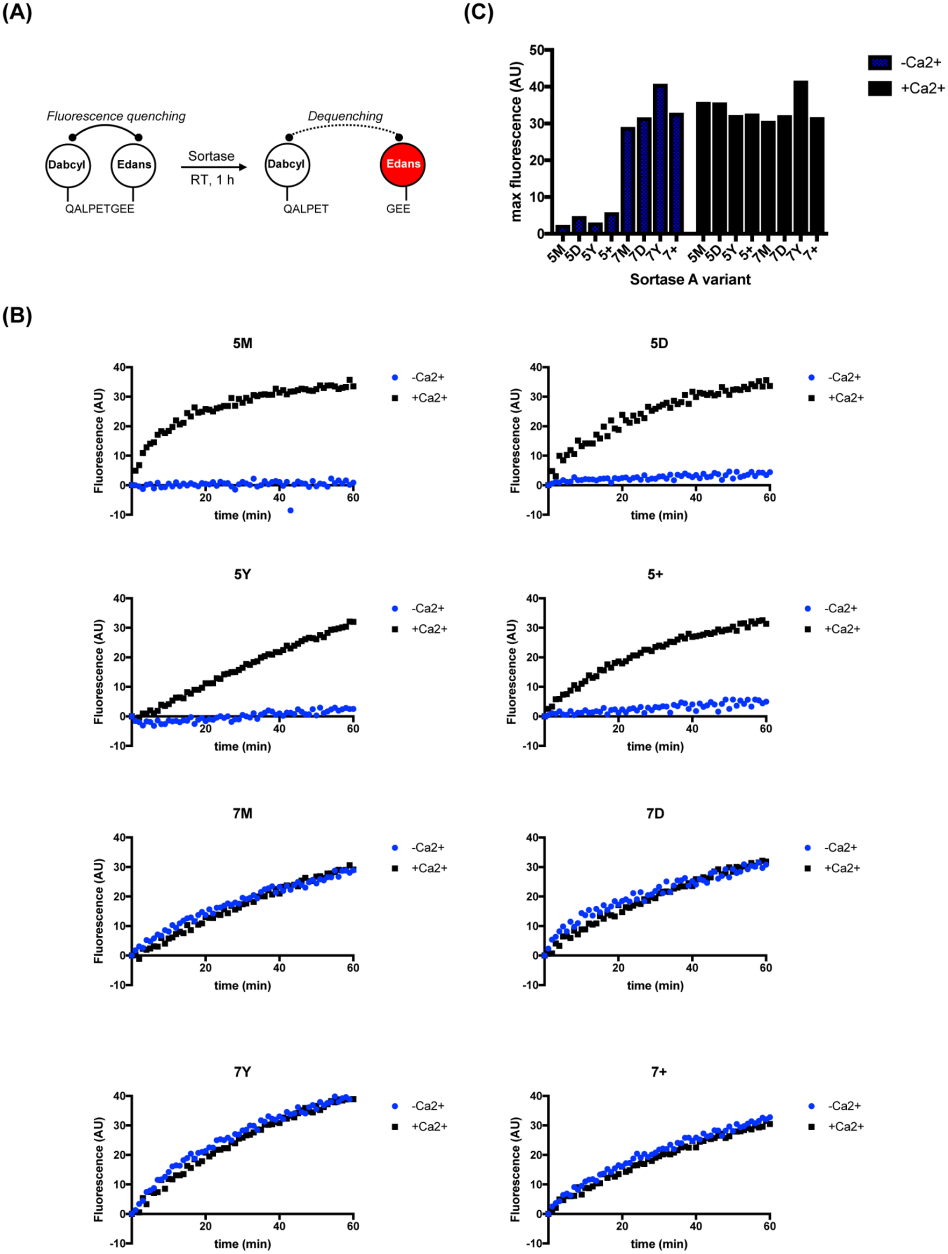
7X SrtA all have catalytic activity. (A) Schematic representation of a FRET-based cleavage assay that measures the initial cleavage of the Thr-Gly scissile bond of Dabcyl-QALPETGEE-Edans peptide. (B) 5 μM of each Srt A variants and 40 μM substrate were incubated in the presence or absence of Ca^2+^, and substrate cleavage was monitored by an increase in fluorescence (arbitrary units). (C) Maximum fluorescence intensity reached for each variant in one hour at room temperature. Assays were performed in duplicate and averaged.

We first tested both the previously reported and newly constructed sortase A variants’ ability to cleave a model substrate (Dabcyl-QALPETGEE-Edans) using a standard, plate-based FRET assay, in both the presence and absence of 10mM CaCl_2_ [23] (Fig 2A). 5X variants only showed significant activity when Ca^2+^ was present. The 7X series’ activities were largely independent of the presence of calcium in the reaction, with the 7Y variant showing the highest activity against the substrate as determined by the maximum fluorescence measured after a one-hour incubation at room temperature (Fig 2B and 2C).

### 7X SrtA mutants demonstrate calcium-independent activity

**Fig 3:**
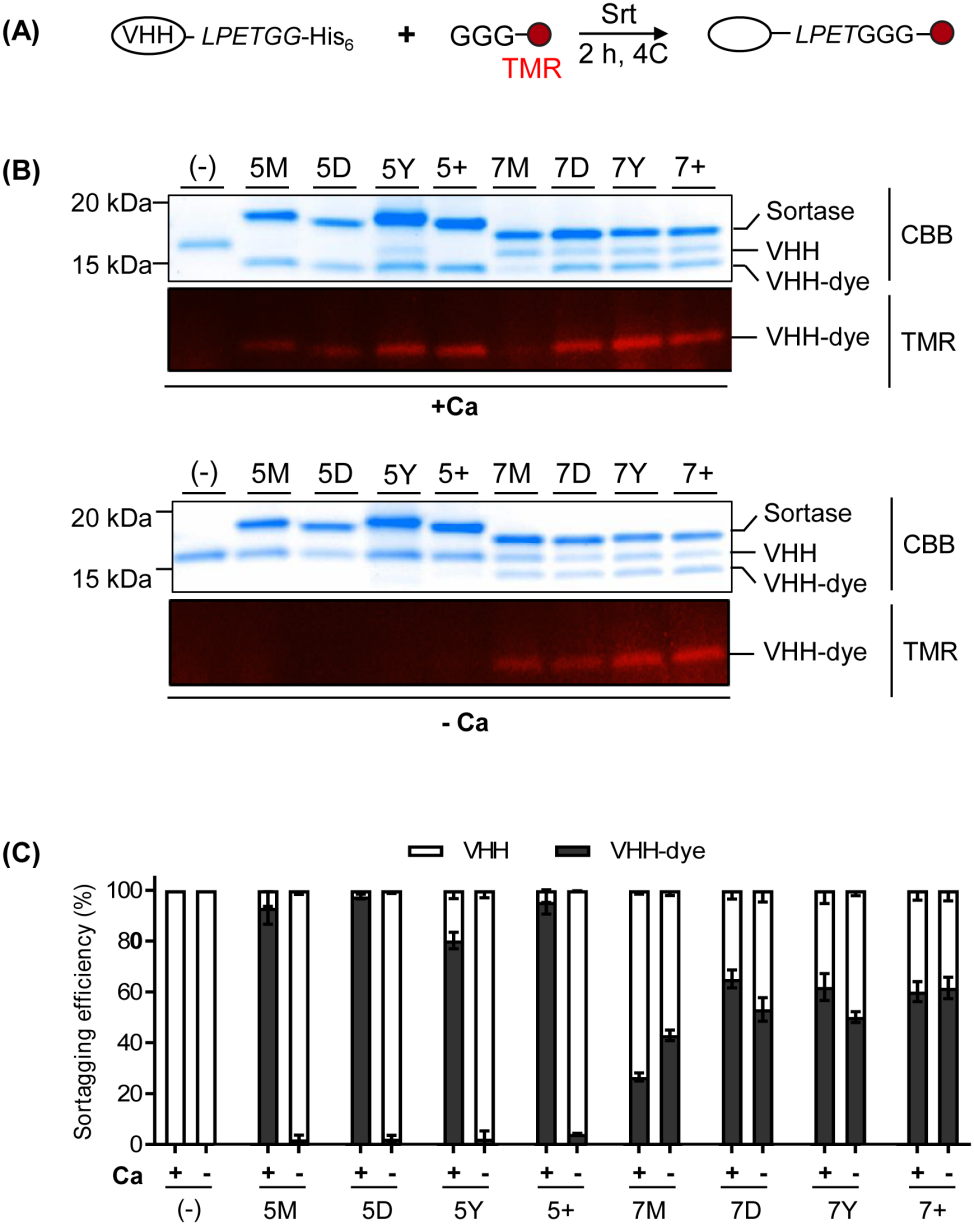
7X SrtA variants show calcium-independent C-terminal sortagging efficiency. (A) Schematic representation of C-terminal fluorescent labeling of a VHH using SrtA. LPETGG-conjugated VHH1 and GGG-conjugated TAMRA were incubated with SrtA variants in buffer containing 10 mM CaCl_2_ or 10 mM EGTA at 4°C for 2 h. (B) Coomassie-stained SDS-PAGE gel and its corresponding fluorescent image with a 488 nm emission filter (C) Sortagging efficiency was quantified for each of these conditions based on relative band intensity between dye-labeled VHH1 (lower Coomassie-stained band) and unlabeled VHH1 (upper Coomassie-stained band). Error bars represent ±1 standard deviation (SD) (n = 3).

Since the FRET assay only measures initial disruption of the Thr-Gly scissile bond in the substrate peptide, and not transpeptidation for lack of a (Gly)n nucleophile, we next tested the variants’ ability to fluorescently label a model protein substrate in the presence or absence of 10mM CaCl_2_. We chose single domain antibody fragments derived from heavy chain only antibodies of alpacas (also termed VHHs, or nanobodies) as protein substrates due to their ease of expression in *E*.*coli*, their high stability and solubility, and their ability to tolerate modification at both the N and C-termini [24,25]. For C-terminal sortagging experiments, we used a previously reported nanobody, DC15, which serves as the model substrate we here refer to as VHH1, and which bears a C-terminal LPETG motif (S1 Fig)[26]. For each reaction, 5 μM VHH1-LPETGG, 500 μM GGGK-TAMRA and 5 μM of each of the SrtA mutants was incubated in the presence or absence of 10mM CaCl_2_ (Fig 3A). All eight SrtA variants successfully conjugated dye to the VHH, with 5M, 5D, and 5+ SrtA yielding the highest overall efficiency when calcium was present in the reaction. Only the 7X SrtA mutants (7M, 7D, 7Y, 7+) resulted in successful VHH-dye conjugation in the absence of Ca^2+^, although the overall incorporation of label was 40–60% less than that of the 5X SrtA variants in the presence of 10mM CaCl_2_ (Fig 3B and 3C).

**Fig 4:**
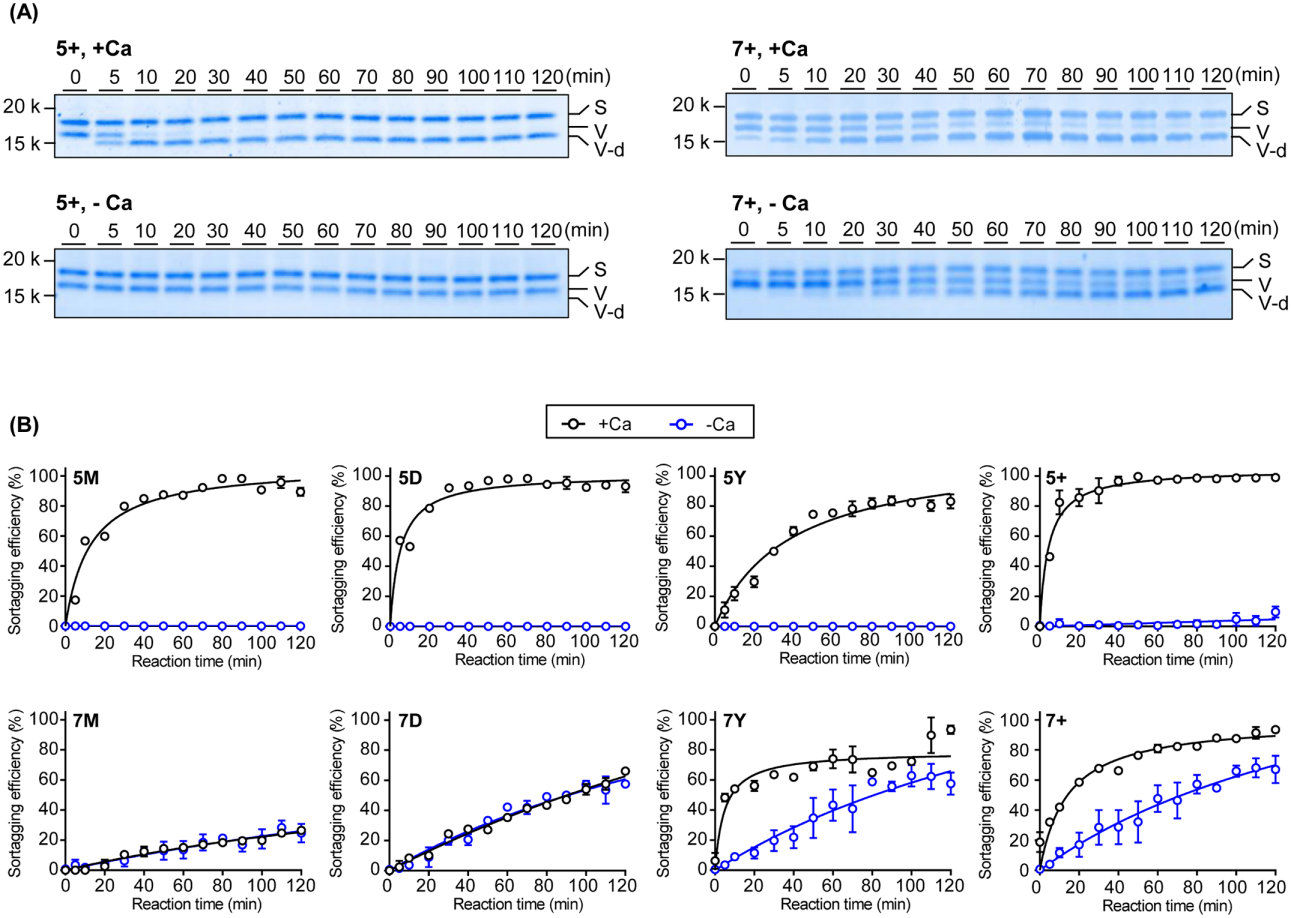
7X SrtA variants show calcium-independent N-terminal sortagging efficiency. (A) Schematic representation of N-terminal fluorescent labeling of protein using sortase. GGGGG-conjugated VHH2 and LPETGG-conjugated fluorescence dye (Alexa647) were incubated with sortase in buffer containing 10 mM CaCl_2_ or 10 mM EGTA at 4°C for 2 h. (B) Coomassie stained SDS-PAGE gel and its corresponding fluorescence image with a 647 nm emission filter.

Similar to our C-terminal sortagging experiments, we determined the efficacy of N-terminal sortagging of the mutants by testing their ability to label VHH2, another previously published VHH against mouse PD-L1 that bears an N-terminal GGGGG extension (S1 Fig) [27]. For each reaction, 5 μM GGGGG-VHH2, 500 μM AlexaFluor647-LPETGG and 5 μM of each of SrtA mutant was incubated in the presence or absence of 10mM CaCl_2_ (Fig 4A). Again all eight SrtA variants conjugated VHH to dye in the presence of 10mM CaCl_2_, but only the 7X SrtA variants and—to a lesser extent—the 5+ SrtA mutant, were active in the absence of Ca^2+^ (Fig 4B).

### 7+ SrtA shows enhanced activity both in the presence and absence of calcium

**Fig 5:**
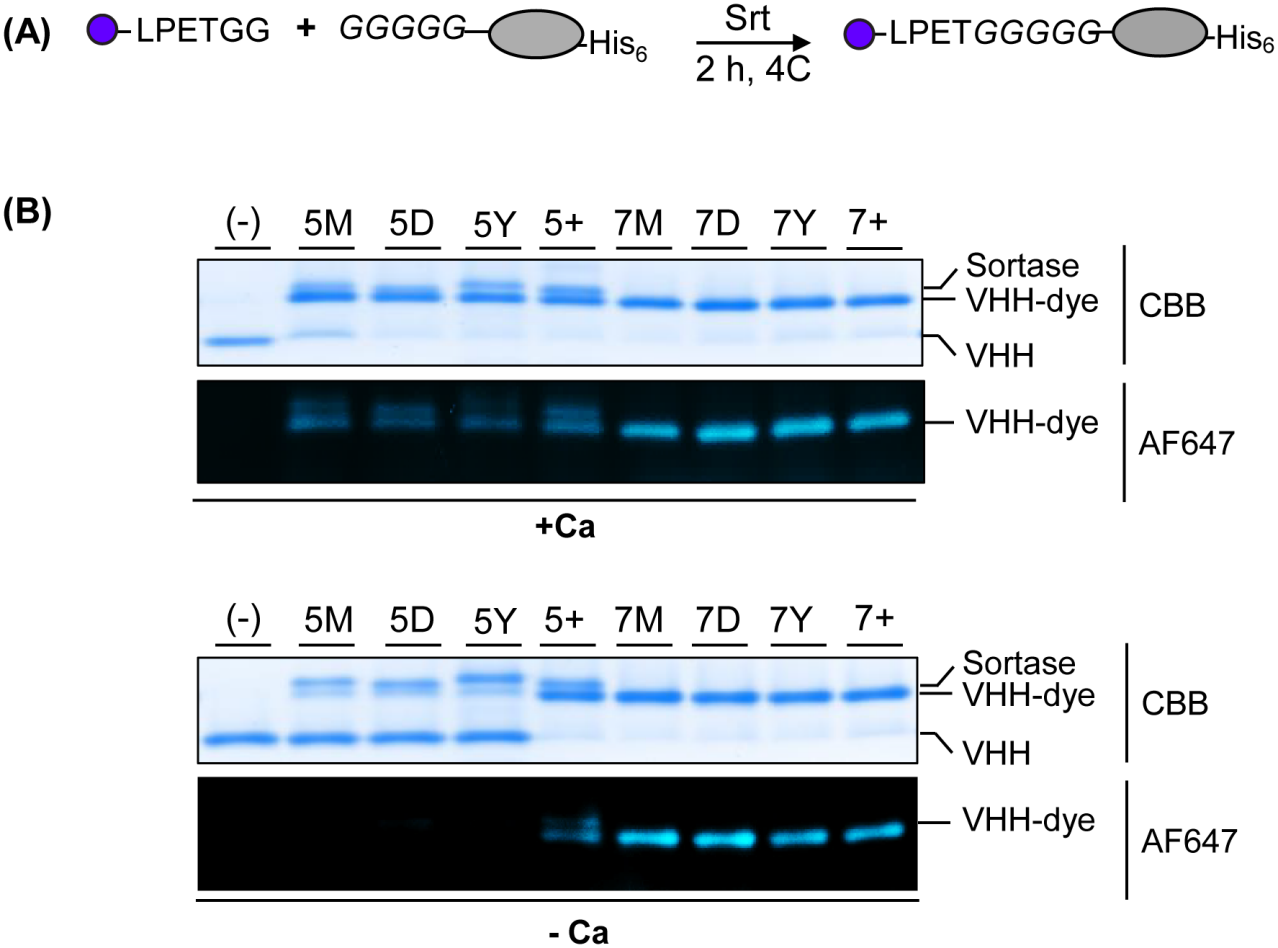
SrtA variants show variable labeling activities in the presence or absence of Ca^2+^. (A) 5+ SrtA (left) or 7+ SrtA (right) was incubated with LPETGG-conjugated DC15 and GGG-conjugated TAMRA in buffer containing 10 mM CaCl_2_ or EGTA at 4°C for various time points (0, 5, and 10 minute intervals up to 2 h). The reaction was stopped by denaturation with SDS-loading buffer. Bands indicate sortase (S), VHH1 (V) and VHH1-dye (V-d). (B) Sortagging efficiency of each sortase variant in the presence or absence of Ca^2+^ over time up to 2 h. Sortagging efficiency was quantified based on relative band intensity between dye-labeled VHH1 and unlabeled VHH1. Error bars represent ±1 SD (n = 3).

To determine whether there were differences in the rates of sortagging among the variants, we incubated 5 μM VHH1-LPETGG, 500 μM GGGK-TAMRA, and 5 μM of each SrtA variant in the presence or absence of 10mM CaCl_2_ for a time course of up to two hours at 4°C. The 5X SrtA mutants (5M, 5D, 5Y, 5+) showed no activity in the absence of calcium. In the presence of calcium, all 5X mutants exhibited similar rates of product formation. 5+ SrtA showed an enhanced rate of product formation, reaching saturation by 20 minutes, while 5Y exhibited a shallower curve, the reaction not reaching completion until after the 1-hour time point. 7M and 7D SrtA showed identical rates at 0 or 10mM CaCl_2_. In contrast, reactions with 7Y and 7+ SrtA variants occurred at faster rates in the presence of calcium, more akin to the 5X mutants, and with slower rates in the absence of calcium (Fig 5). These time-course experiments suggest that a longer incubation time, e.g. 2 hours, or possibly increased temperature, is preferable when using the 7X mutants in low calcium concentrations.

**Fig 6:**
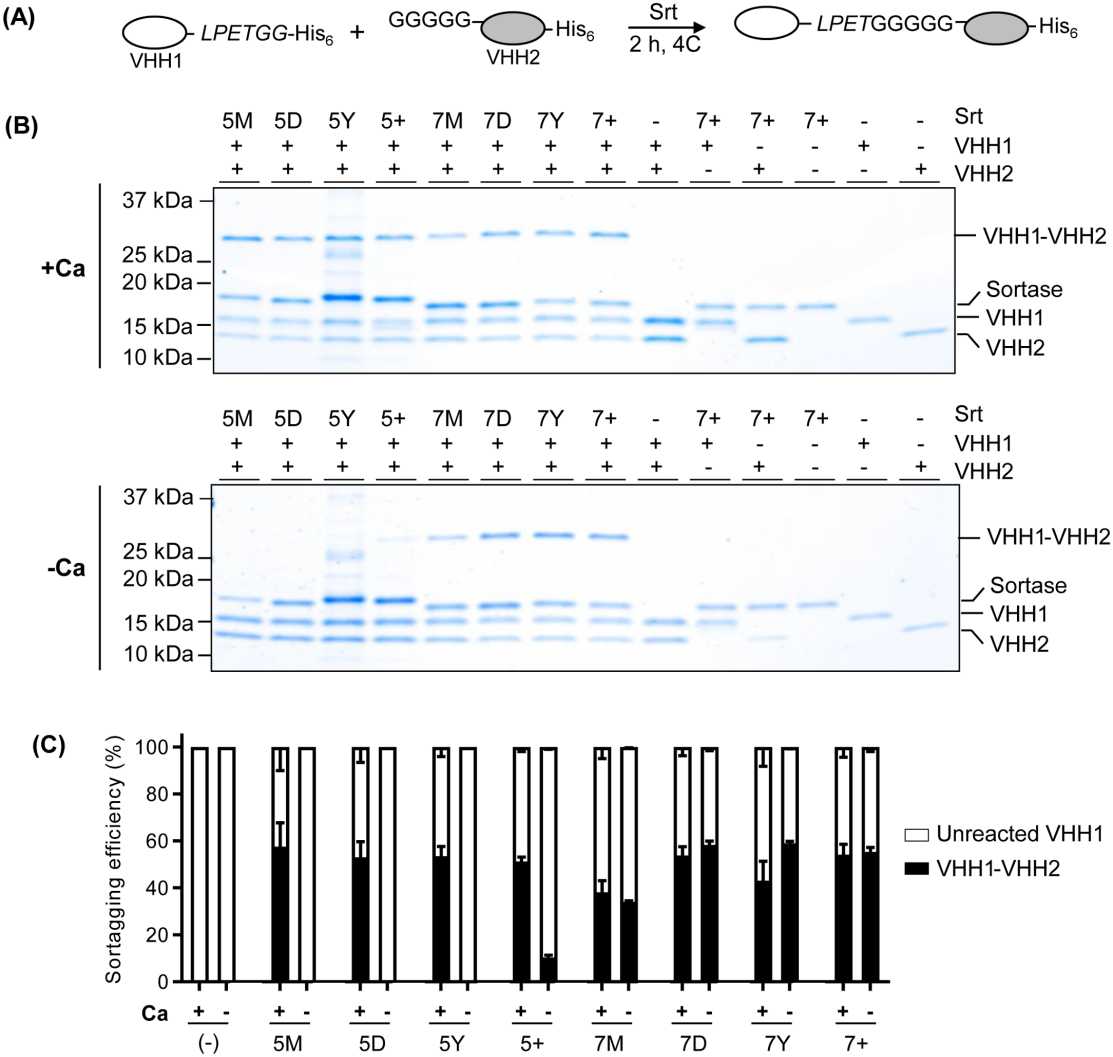
Calcium-independent protein-protein conjugation using 7X SrtA variants. (A) Schematic representation of protein-protein conjugation using sortase. LPETGG-conjugated VHH1 and GGGGG-conjugated VHH2 were incubated with sortase in buffer containing 10 mM CaCl_2_ or EGTA at 4°C for 2 h. (B) The bands indicated are conjugated VHH1-VHH2, sortase, unconjugated VHH1, and unreacted VHH2 after sortagging with Ca^2+^ (top) or without Ca^2+^ (bottom). (C) Sortagging efficiency was quantified for each of these conditions based on relative band intensity between conjugated VHH1-VHH2 and unlabeled VHH1. Error bars represent ±1 standard deviation (SD) (n = 3).

We then analyzed the efficiency of protein-protein conjugation by the various sortase mutants. To model protein-protein conjugation, we chose to conjugate two VHHs together: VHH1-LPETG and GGGGG-extended VHH2 (Fig 6A). The formation of a 29kDa VHH1-VHH2 product indicates successful protein conjugation (Fig 6B). As expected, the 5X SrtA mutants showed calcium-dependent protein conjugation, while the 7X mutants were calcium-independent (Fig 6C). In the absence of calcium, 7D, 7Y, and 7+ SrtA were equally efficient; in the presence of calcium, all of the 5X SrtA variants were equivalent.

### 7+ SrtA is the optimal variant for cell surface labeling applications

**Fig 7:**
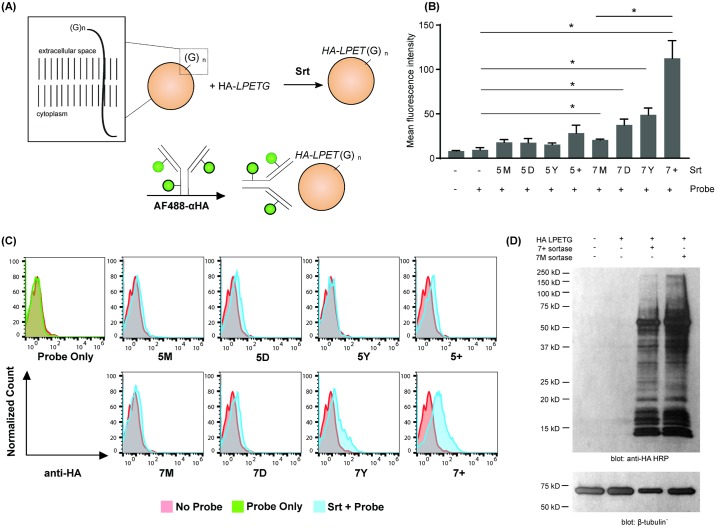
Cell surface labeling using SrtA variants. (A) Schematic representation of lymphocyte surface labeling. Sortase A labels cell populations with covalently linking a HA-LPETG probe to N-terminal oligoglycines on the cell surface. (B) The efficacy of cell surface labeling by the different SrtA mutants was measured by flow cytometry. Representative plots are shown of the mean fluorescence intensity (MFI) of anti-HA AlexaFluor488 with or without sortase A. Error bars represent ±1 SD (n = 3). Statistical analysis was performed using unpaired t test with Welch’s correction. The asterisks indicate p<0.05 for the indicated SrtA mutant as compared to the “probe only” control. Labeling using 7M was significantly higher than probe only (p = 0.0342) and 7+ performed significantly better than 7M (p = 0.0434). (C) Representative overlays of normalized cell count histograms showing AF488 anti-HA labeling intensity on lymphocytes incubated with HA-LPETG probe and SrtA (blue), probe only (green), or without either SrtA or probe (red). (D) Conjugation of the HA-LPETG probe to the cell surface by 7+ SrtA and 7M SrtA was verified by western blot using anti-HA-HRP antibody. Anti-beta-tubulin-HRP was used as a loading control.

Most cells, including murine lymphocytes, naturally express surface-exposed N-terminal glycines, which can serve as nucleophiles in sortase reactions [18]. In order to determine the efficacy of cell surface labeling, we incubated mouse spleen cells with SrtA variants and a HA-LPETG substrate in media supplemented with 10mM CaCl_2_ (Fig 7). While all of the SrtA variants labeled the cell surface to some degree, 7+ SrtA showed the greatest efficacy in our assay, with a 6-fold higher activity than 5M SrtA. The 5+, 7D, 7M and 7Y SrtA variants also resulted in a 2-fold increase in cell surface labeling over the control. Western blot analysis confirmed that the HA staining identified by flow cytometry was due to conjugation of HA-LPETG, and not non-specific binding of peptide or the peptide-SrtA conjugate, to the cell surface (Fig 7D). We conclude that the 7+ SrtA mutant is optimal for such cell surface labeling applications. Given its reduced reliance on Ca^2+^, 7+ SrtA is likely to be able to label the cell surface over a wide physiological range of Ca^2+^ concentrations.

## Discussion

In this study, we combined previously described mutations to generate new, calcium-independent sortase variants. These variants are active at both 0 and 10mM CaCl_2_ in applications such as N and C-terminal protein labeling and protein-protein conjugation, although the activity of Ca^2+^-independent variants was generally lower in the absence of added calcium. Our experiments converged on a variant (7+ SrtA) as the enzyme with optimal performance in cell surface labeling applications.

We performed *in vitro* labeling experiments under conditions likely to be amenable to a broad range of substrates, i.e. low temperatures and short incubation times, although it is likely that parameters such as amount of enzyme, temperature and length of incubation will need to be optimized for each substrate:nucleophile pair. Sortase enzymes have also been reported to accept bivalent cations other than Ca^2+^ as cofactors, and such information may prove useful particularly for *in vivo* sortagging applications, although carefully analysis of cofactor use was beyond the scope of this study [28].

The 7+ SrtA surface-labeled cells more efficiently than previously described sortase variants [18]. The mutations in the 5+ variant appear to affect the flexibility of loops within the substrate binding region as previously described [10]. When combined with mutations at E105 and E108 that confer calcium independence, these mutations continue to enhance activity, but the interactions between the composite mutations need to be further explored at atomic resolution to better understand why no variants maintain full activity in the absence of calcium.

The use of sortase for *in vivo* protein ligation enables creation of non-genetically encoded, site-specific and covalent linkages under a variety of conditions. In addition to *ex vivo* or *in vivo* cell surface labeling, 7+ SrtA may also be useful for other calcium-sensitive applications, including intracellular sortagging, as the Ca^2+^ concentration in the cytoplasm of living cells would render calcium-dependent SrtA variants inactive in this environment.

We view the present work as a logical next step towards improving SrtA for cell-based applications. In this general area, SrtA-mediated transacylations must compete with several other methods, each with its signature advantages. The enzyme butelase recognizes a three-residue motif, LHV, accepts a wide range of incoming nucleophiles, and has an impressive K_cat_, but a drawback of butelase is the inability to produce it in active form recombinantly [29]. Formylglycine generating enzyme (FGE) has the advantage of being able to target cysteine residues regardless of their position in the polypeptide chain, as long as the residue is accessible. For secretory and membrane proteins, the simultaneous presence of disulfide bonds and an unpaired cysteine require careful consideration when using FGE [30]. Biotin ligase, arguably an early arrival on the scene of chemo-enzymatic transformations, shares with SrtA the advantage of a rather short recognition motif and pronounced substrate specificity [31]. However, the synthesis of biotin derivatives is not without its challenges. These factors help explain the increasing acceptance of SrtA and its derivatives as a convenient method for protein modification with a wide range of substituents. The SrtA variants described here will enable more creative cell biological applications.

## Supporting information

S1 Fig Generation and purification of VHHs. SDS-PAGE gel of bacterially expressed VHHs following NiNTA purification. VHH1 (“DC15”) bears a C-terminal LPETGG and VHH2 (“A12”) bears an N-terminal GGGGG extension[26,27].
(TIF)

S1 Table Amino acid sequences, molecular weights and extinction coefficients of proteins used in this study. (DOCX)
