# Supplementary figures and images for: Generation of Ca2+-independent sortase A mutants with enhanced activity for protein and cell surface labeling
Source: PLoS One. 2017 Dec 4;12(12):e0189068. doi: 10.1371/journal.pone.0189068 (PMC5714338; doi:10.1371/journal.pone.0189068)

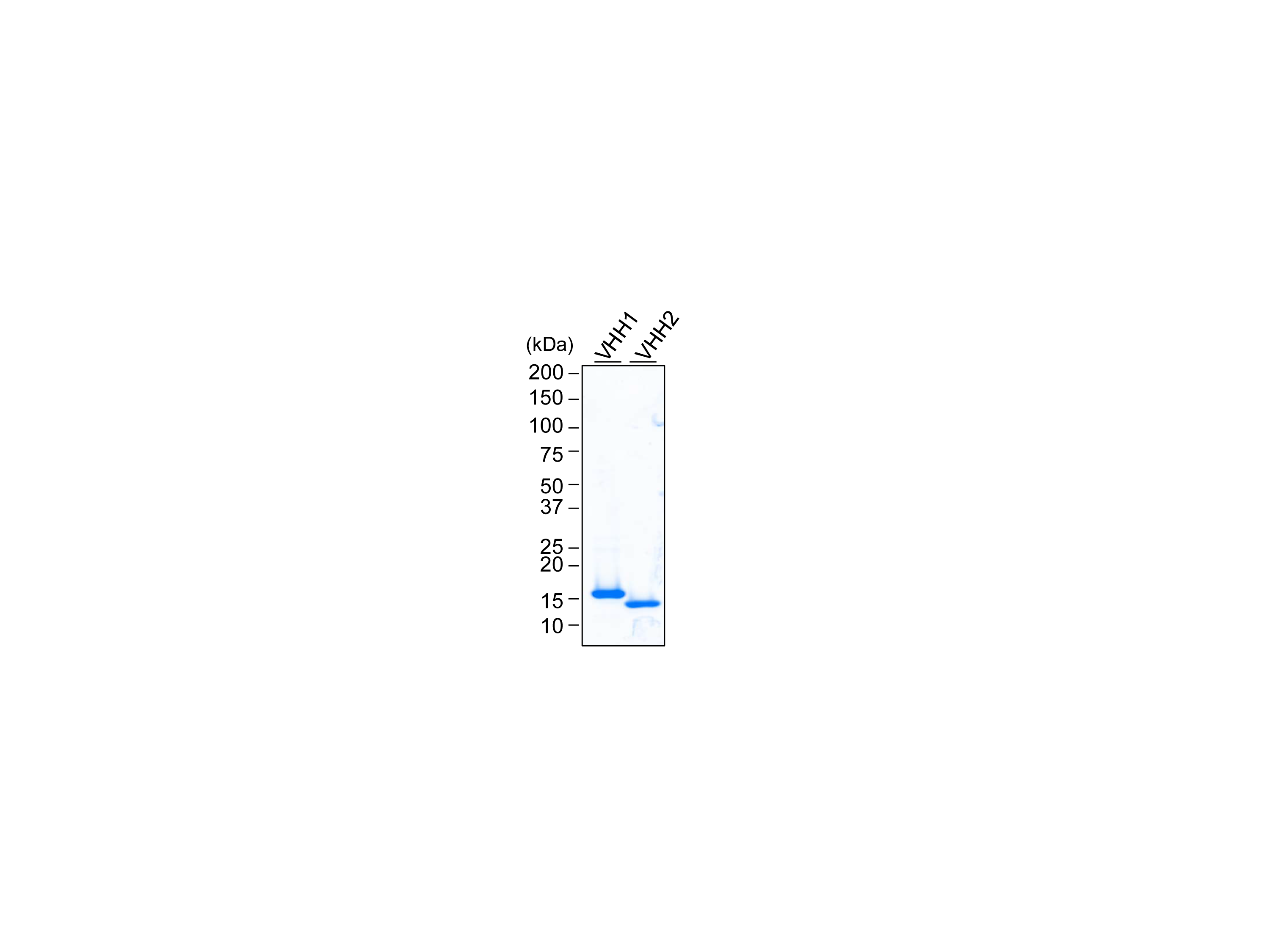

Supplement: S1 Fig — SDS-PAGE gel of bacterially expressed VHHs following NiNTA purification. VHH1 (“DC15”) bears a C-terminal LPETGG and VHH2 (“A12”) bears an N-terminal GGGGG extension[26,27]. (TIF) [file pone.0189068.s001.tif]
