# Supplementary material for: Generation of Ca2+-independent sortase A mutants with enhanced activity for protein and cell surface labeling
Source: PLoS One. 2017 Dec 4;12(12):e0189068. doi: 10.1371/journal.pone.0189068 (PMC5714338; doi:10.1371/journal.pone.0189068)
Supplement: S1 Table — (DOCX) [file pone.0189068.s002.docx]

|  | Amino acid sequence | MW (kDa) | EC |
| --- | --- | --- | --- |
| 5M | MQAKPQIPKDKSKVAGYIEIPDADIKEPVYPGPATREQLNRGVSFAEENESLDDQNISIAGHTFIDRPNYQFTNLKAAKKGSMVYFKVGNETRKYKMTSIRNVKPTAVEVLDEQKGKDKQLTLITCDDYNEETGVWETRKIFVATEVKLEHHHHHH | 17.85 | 14440 |
| 5D | MQAKPQIPKDKSKVAGYIEIPDADIKEPVYPGPATREQLNRGVSFAEENESLDDQNISIAGHTFIGRPNYQFTNLKAAKKGSMVYFKVGNETRKYKMTSIRNVKPTAVEVLDEQKGKDKQLTLITCDDYNEETGVWETRKIFVATEVKLEHHHHHH | 17.80 | 14440 |
| 5Y | MQAKPQIPKDKSKVAGYIEIPDADIKEPVYPGPATREQLNRGVSFAEENESLDDQNISIAGHTFIDRPNYQFTNLKAAKKGSMVYFKVGNETRKYKMTSIRNVKPTAVEVLDEQKGKDKQLTLITCDDLNRETGVWETRKIFVATEVKLEHHHHHH | 17.83 | 12950 |
| 5+ | MQAKPQIPKDKSKVAGYIEIPDADIKEPVYPGPATREQLNRGVSFAEENESLDDQNISIAGHTFIGRPNYQFTNLKAAKKGSMVYFKVGNETRKYKMTSIRNVKPTAVEVLDEQKGKDKQLTLITCDDLNRETGVWETRKIFVATEVKLEHHHHHH | 17.77 | 12950 |
| 7M | MQAKPQIPKDKSKVAGYIEIPDADIKEPVYPGPATREQLNRGVSFAKENQSLDDQNISIAGHTFIDRPNYQFTNLKAAKKGSMVYFKVGNETRKYKMTSIRNVKPTAVEVLDEQKGKDKQLTLITCDDYNEETGVWETRKIFVATEVKLEHHHHHH | 17.85 | 14440 |
| 7D | MQAKPQIPKDKSKVAGYIEIPDADIKEPVYPGPATREQLNRGVSFAKENQSLDDQNISIAGHTFIGRPNYQFTNLKAAKKGSMVYFKVGNETRKYKMTSIRNVKPTAVEVLDEQKGKDKQLTLITCDDYNEETGVWETRKIFVATEVKLEHHHHHH | 17.79 | 14440 |
| 7Y | MQAKPQIPKDKSKVAGYIEIPDADIKEPVYPGPATREQLNRGVSFAKENQSLDDQNISIAGHTFIDRPNYQFTNLKAAKKGSMVYFKVGNETRKYKMTSIRNVKPTAVEVLDEQKGKDKQLTLITCDDLNRETGVWETRKIFVATEVKLEHHHHHH | 17.83 | 12950 |
| 7+ | MQAKPQIPKDKSKVAGYIEIPDADIKEPVYPGPATREQLNRGVSFAKENQSLDDQNISIAGHTFIGRPNYQFTNLKAAKKGSMVYFKVGNETRKYKMTSIRNVKPTAVEVLDEQKGKDKQLTLITCDDLNRETGVWETRKIFVATEVKLEHHHHHH | 17.77 | 12950 |
